# Supplementary material for: Choice of intraoperative ultrasound adjuncts for brain tumor surgery
Source: BMC Med Inform Decis Mak. 2022 Nov 28;22:307. doi: 10.1186/s12911-022-02046-7 (PMC9703786; doi:10.1186/s12911-022-02046-7)
Supplement: Supplementary file 1 — Additional file 1. Correlation matrices and details of the redesigned variables. [file 12911_2022_2046_MOESM1_ESM.pdf]

# 1 Supplement

## 1.1 Correlation Matrices

| Name | Gender | Surgeon Exp | Prior treatment | Contrast Enhancement pattern | Delineation | Eloquent | Histology | Depth of tumor | Length | Width | Height | Glioma Location |
|---|---|---|---|---|---|---|---|---|---|---|---|---|
| **Age** | 0.035 | 0.09 | -0.22 | 0.164 | -0.108 | -0.01 | 0.28 | 0.027 | -0.126 | -0.188 | -0.126 | 0.262 |
| **Gender** | | 0.05 | -0.04 | -0.001 | 0.00 | 0.01 | 0.00 | 0.00 | -0.017 | -0.039 | 0.013 | -0.019 |
| **Surgeon Exp** | | | -0.08 | -0.019 | -0.01 | 0.08 | 0.06 | -0.022 | -0.032 | -0.057 | 0.013 | 0.034 |
| **Prior treatment** | | | | 0.0 | 0.00 | -0.03 | 0.00 | -0.002 | -0.048 | -0.035 | -0.04 | 0.006 |
| **Contrast Enhancement pattern** | | | | | 0.24 | 0.00 | 0.15 | -0.019 | -0.078 | 0.012 | -0.042 | -0.002 |
| **Delineation** | | | | | | 0.0 | -0.08 | -0.152 | -0.016 | -0.002 | 0.007 | -0.002 |
| **Eloquent** | | | | | | | 0.0 | -0.002 | 0.007 | 0.087 | 0.08 | -0.019 |
| **Histology** | | | | | | | | 0.006 | -0.079 | -0.078 | -0.092 | -0.005 |
| **depth of tumor** | | | | | | | | | -0.116 | -0.137 | -0.159 | 0.002 |
| **length** | | | | | | | | | | **0.697** | **0.683** | 0.013 |
| **width** | | | | | | | | | | | **0.667** | -0.102 |
| **Height** | | | | | | | | | | | | -0.024 |

Table 1: Correlation computed using different methods for categorical, ordinal, and continuous variables.

| Name | Gender | Surgeon Exp | prior treatment | Contrast Enhancement pattern | Delineation | Eloquent | Histology | Depth of tumor | Spherical Diameter | Glioma Location |
|---|---|---|---|---|---|---|---|---|---|---|
| **Age** | 0.035 | 0.09 | -0.22 | 0.164 | -0.108 | -0.01 | 0.28 | 0.027 | -0.158 | 0.262 |
| **Gender** | | 0.05 | -0.04 | -0.001 | 0.00 | 0.01 | 0.00 | 0.00 | -0.017 | -0.019 |
| **Surgeon Exp** | | | -0.08 | -0.019 | -0.01 | 0.08 | 0.06 | -0.022 | -0.035 | 0.034 |
| **prior treatment** | | | | 0.00 | 0.00 | -0.03 | 0.00 | -0.002 | -0.047 | 0.006 |
| **Contrast Enhancement pattern** | | | | | 0.24 | 0.00 | 0.15 | -0.019 | 0.039 | -0.002 |
| **Delineation** | | | | | | 0.00 | -0.08 | -0.152 | -0.006 | -0.002 |
| **Eloquent** | | | | | | | 0.00 | -0.002 | 0.065 | -0.019 |
| **Histology** | | | | | | | | 0.006 | -0.089 | -0.005 |
| **depth of tumor** | | | | | | | | | -0.157 | 0.002 |
| **Spherical Diameter** | | | | | | | | | | -0.041 |

Table 2: Correlation matrix with spherical diameter.

## 1.2 Statistical Analysis of data set of refined variables

| | | **2D (n=143)** | **Navigated 3D (n=207)** | **P-value‡** |
|---|---|---|---|---|
| Prior Treatment | No | 98 | 171 | |
| | Yes | 45 | 36 | 0.002* |
| Contrast Enhancement Pattern | Non-Enhancing | 33 | 78 | |
| | Enhancing | 110 | 129 | 0.004* |
| Delineation (PMD) | Poor | 90 | 125 | |
| | Good | 53 | 82 | 0.63 |
| Delineation (GMD) | Poor | 10 | 23 | |
| | Good | 133 | 184 | 0.19 |
| Eloquent Location | No | 88 | 109 | |
| | Yes | 55 | 98 | 0.10 |
| Location | Superficial | 103 | 134 | |
| | Deep | 40 | 73 | 0.66 |
| Histology | Low grade | 31 | 49 | |
| | High grade | 112 | 158 | 0.15 |

Table 3: Distribution of data after converting ordinal variables to dichotomous. * denotes $p < 0.05$, p-values corresponding to Mann-Whitney test.
